# Supplementary material for: Global, selective, or both? The case for differentiated cooperation in AI governance
Source: Policy Sci. 2026 Mar 9;59(1):203–23. doi: 10.1007/s11077-026-09606-y (PMC13031221; doi:10.1007/s11077-026-09606-y)
Supplement: Supplementary file 1 — Supplementary Material 1 [file 11077_2026_9606_MOESM1_ESM.docx]

# Global, Selective, or Both?

# The Case for Differentiated Cooperation in AI Governance

# *Abstract*

*Current debates about international cooperation in AI governance remain both simplistic and muddled, pitting global forms of collaboration against selective alliances among “like-minded countries”. We propose a more nuanced and systematic approach to cooperative AI governance based on three considerations. First, different kinds of governance issues lend themselves to different kinds of cooperation. Second, not all AI is created equally: different kinds of AI raise different governance challenges, thus requiring varied forms of cooperation. Third, the same is true for the* development *and* deployment *phases of AI systems. Integrating these three factors, we explain why some challenges can only be addressed through global cooperation, while for others selective cooperation is an equally effective, or even more effective, solution. In consequence, governments should not insist on either global or selective cooperation. Instead, they should opt for the scope of collaboration that is most effective for solving any particular governance issue at hand.*

**Keywords:** Artificial intelligence; governance; international cooperation; alliance-building; regulation

# Introduction

The opportunities and challenges created by AI have inspired governments to intervene in its development and deployment. Beyond unilateral efforts—in which we include EU-level initiatives—jurisdictions have launched international cooperation in AI governance, for example through the Organization for Economic Cooperation and Development (OECD) or the United Nations (UN).

Such cooperation remains contested. Some policy and academic analyses claim that effective AI governance requires global cooperation, *tout court.* In principle, such initiatives could be combined with selective cooperation at the regional level. But some argue that a global “focal organization” (Büthe and Mattli 2011) for AI governance is necessary (Hoos and Irgens 2023), subsuming many of the already existing, smaller-scale AI governance initiatives. Thus, in its 2023 interim report, the UN High-Level Advisory Body on Artificial Intelligence declared categorically that “AI governance must be universal” and that “new horizontal coordination and supervisory functions are required and they should be entrusted to a new organizational structure” (UN High-Level Advisory Body on Artificial Intelligence 2023, p.14-16).^[[1]](#footnote-1)^

Plans to make the UN Secretariat the superstructure for AI governance, contained in a leaked 2024 draft report of the UN AI Advisory Body, quickly elicited a backlash (Global Partners Digital 2024; Kerry 2024; Kerry et al. 2024; Kratsios 2025), and Western think tanks and research institutes have warned that cooperation with authoritarian countries—especially China—on digital governance rules would be undesirable (Allen 2022; Cordell 2020). Both US and European policymakers frequently cite value clashes as reasons why democracies cannot collaborate with non-democracies on AI rules (Author, 2023). Taken together, the discussion pits selective against global cooperation as mutually exclusive approaches.

To our mind, this debate is too simplistic for three reasons. First, different kinds of governance issues lend themselves to different kinds of cooperation. For example, *a priori*, global public goods problems are in many cases best dealt with by international organizations with (nearly) global membership, while military alliance building by its very nature requires selective cooperation within smaller organizations. Second, the AI moniker subsumes such diverse digital systems that different cooperation rationales for them—stemming from the different governance issues they pose—are only to be expected. Large language models (LLMs), for example, raise very different challenges than AI-powered home appliances. Since the issues that need governing in the case of AI vary enormously (Mueller 2024; Nitzberg and Zysman 2022; Taeihagh 2021), so do the rationales for collaborating and for embracing one or the other scope of collaboration (cf. Beaumier et al. 2020). And third, governance challenges—and hence incentives for cooperation and the most effective ways to do so—vary significantly between the *development* phase of an AI system and its subsequent *deployment*. States may therefore need to prioritize one kind of cooperation in the development phase and another in the deployment phase. Against that background, we argue, cooperation in AI governance needs to be approached differently: when and where is global cooperation necessary to tackle governance challenges, and when is selective cooperation an equally viable (or even the better) solution?

To answer this question, we use a deductive approach, marrying theories of cooperation and typologies of collectively produced goods to an understanding of the diverse challenges AI can pose. We use this approach for two reasons: first, much of the debate is about the potential and desirability of cooperation, given that cooperative AI governance is still nascent and the empirical track record therefore thin. Second, the deductive approach allows us to isolate the dynamics we are interested in here—the problem-solving potential of different forms of cooperation—from other factors such as rising techno-nationalism, which confound observable patterns of cooperation in AI governance (Justo-Hanani 2022).

In a first step, we disentangle potential goals of cooperative AI governance. We then build a framework to assess the effectiveness of selective and global cooperation with reference to each of those goals. That allows us to see when global cooperation is necessary—and when selective cooperation is an equally or even more effective solution. While private actors can—and do—play an important role in AI governance, we concentrate on state actions, as only states and multilateral bodies can engage in most forms of cooperation relevant here, such as crafting international law or building geopolitical alliances.

In total, we analyze seven (intermediate) goals for interstate cooperation on AI governance, drawing on theories in International Relations (IR) and International Political Economy (IPE): building or bolstering geopolitical alliances; avoiding global arms races/races to the bottom; preventing the proliferation of harmful AI; realizing benefits from trade; sharing knowledge and best practices; promoting norms; managing externalities of harmful AI, and collective action problems. Because many of these issues can be tackled independently from each other, countries need not choose between the two approaches to AI governance. Instead, a *differentiated* approach to cooperation makes much more sense.

Figuring out where selective cooperation in AI governance is most effective and where global cooperation is the better (or maybe the only) option is important for two reasons. From an analytical perspective, it helps us understand AI governance challenges better, including which of them are most salient in which phase of the AI lifecycle, and through which governance mode governments can best address them. From a practical perspective, our arguments help policymakers decide when to pursue global AI cooperation, and when to aim for selective cooperation instead.

Other domains of global governance have shown how such a variegated perspective can expand the possibilities for cooperation, lending additional support to the deductive approach we take here. The governance of nuclear technologies for example follows a logic similar to the one proposed here. Like AI, nuclear technologies have varied uses in both civilian and military contexts. For public policymakers weighing global against selective cooperation, it therefore matters (1) what the specific goal of cooperation is, (2) what kind of nuclear technology exactly it aims to govern, and (3) whether it aims to intervene in the development or deployment phase. This mirrors the three dimensions shaping the effectiveness of selective and global AI governance, respectively. The nuclear technology regime complex consists of partly overlapping instruments that differ by goal, technology, and timing. Examples range from the near-universal Treaty on the Non-Proliferation of Nuclear Weapons (NPT)/International Atomic Energy Agence (IAEA) safeguards (verification) to selective instruments such as the IAEA Additional Protocol (enhanced verification), the Nuclear Suppliers Group (trade controls), and the Proliferation Security Initiative (operational interdiction) ("Basics of IAEA Safeguards" 2016). This “stack” of agreements allows states to work together on areas in which cooperative preferences align without having to opt into the whole bundle. Analogously, a differentiated approach to AI governance enables states to enter into universal treaties on some issues, while pursuing selective cooperation on others.

This article proceeds as follows: we first delimit the scope of our inquiry and sketch the dominant debate about AI governance. Based on the IR and IPE literature, we then discuss seven intermediate goals for cooperation in AI (that serve larger ultimate goals) and spell out their implications for AI governance challenges. The subsequent section zooms in on one of those categories—externalities from cross-border AI use—and shows how we need to differentiate between the development and deployment phases of the AI life cycle and different forms of AI to pinpoint which kinds of cross-border governance may be needed. The final section combines these elements in a heuristic for understanding AI governance cooperation and spells out how our findings argue in favor of a variable approach to cooperation, rather than one that opts for either the global or the selective variant.

# Delimiting AI Governance

AI is an amorphous and misleading concept. In practice, it has most closely been associated with machine learning (Mitchell 2019), but it also includes various symbolic approaches to building systems that attempt to emulate human thinking (Hitzler *et al.* 2022). We use the label despite this blurriness because it dominates public and policy debates. “AI” structures countless working groups, standard-setting bodies and rule-setting initiatives at both national and international levels (International Telecommunication Union (ITU) 2024). AI is real as a social construction that, as such, leaves a footprint in the world (Searle 1995), including on how the technologies that fall under AI are governed. At the same time, the term injects unhelpful and un-reflected slants into policy debates. The sundry technologies lumped together under the AI heading invite blanket statements about the desirability of “cooperation in AI”—ignoring that automated target identification for military drones is a completely different matter than smart thermostats to keep the kitchen warm. A key part of our mission is to disentangle the AI knot as relevant to governance cooperation, an issue to which we return in the penultimate section of this article.

Our focus makes us pragmatic in how we delimit our field of inquiry. In principle, both public and private actors feature in AI governance. But because we care about interstate cooperation, AI governance here encompasses public actor interventions to shape the development and deployment of AI technologies. Cooperation on AI governance, then, means international coordination of these public interventions.

Cooperation ranges from two jurisdictions working together to something covering the whole globe. Lest matters get unduly complicated, we distinguish two scopes for cooperation here: (more or less) global cooperative AI governance, which certainly includes all the major AI powers (and crucially China and the United States); and limited cooperative AI governance (“selective cooperation”), which includes at least two jurisdictions but misses at least one central AI power.

We observe both global and selective cooperation initiatives in the wild (Author, 2023; Ulnicane 2024), ranging from the UN General Assembly promoting ethical, safe, secure, and trustworthy AI among its 193 member states to transatlantic cooperation through the EU-US Trade and Technology Council (TTC) aimed at fostering democratic approaches to technology. Even though at the time of writing it is unclear whether the TTC will survive in more than form, the transatlantic axis remains a central potential link in global AI governance, considering how the United States and the EU have long stood at the center of multilateral technology governance initiatives.

# Debating Cooperation in AI Governance

Most advocates of cooperation in AI governance fall into one of two camps: some see global cooperation as the only way forward; others champion strategic alliances, instead. Echoing the UN AI Advisory Body’s 2023 call, Chinese foreign minister Wang Yi, too, called for an “international AI governance institution [to] be set up under the UN framework”. Cooperation in a smaller circle of countries (“small yard, high fence,” as he described it) would “result in mistakes with historic consequences” (Yi 2024). Also in academia, we find champions of global AI cooperation. Ala-Pietilä and Smuha (2021, p. 237), for example, find such cooperation necessary to protect citizens, support socially beneficial innovation, and safeguard market competition.

Other scholars and policymakers promote selective cooperation, centered on alliance-building with “like-minded” countries (Kerry *et al.* 2024)—a grouping that typically involves more or less democratic US allies, shutting out others who are thought to have different goals and values (Cheng & Zeng 2023). (We remain agnostic for now here whether those concerns are genuine or only facades to hide hard-nosed geopolitics or economic competition, cf. Paul 2023.) In 2021, the Biden-Harris administration announced its intention to bolster tech cooperation among democracies against authoritarian regimes (White House 2021). Since then, democracies have cooperated on AI standards most meaningfully within the Global Partnership on Artificial Intelligence (GPAI) and the OECD (efforts that effectively merged in 2024), rather than, for example, the UN. GPAI explicitly restricts membership to countries that endorse “Western” values, including respect for human rights, inclusion, and a commitment to democratic principles and international cooperation​. China, so the implication, is not welcome at the table.

And then, there are those who warn that a brewing AI arms race means that anything that slows AI development—including selective cooperation with like-minded countries—only helps the adversary. We find such arguments, for example, from former U.S. Secretary of Defense Mark Esper (Solomon 2023) and former Pentagon Chief Software Officer Nicolas Chaillan (Politi 2021). And in September 2025, Michael Kratsios, Director of the US Office of Science and Technology Policy told the United Nations that

we [the USA] totally reject all efforts by international bodies to assert centralized control and global governance of AI. [..] The path [..] is found not in bureaucratic management, but in the freedom and duty of citizens, the prudence and cooperation of statesmen, and the independence and sovereignty of nations. (Kratsios 2025)

We find, in short, positions ranging from global AI governance as indispensable to somewhere between useless and harmful. But to what degree do these encompassing positions make sense, given the diversity of cooperation incentives, the different challenges raised by different AI lifecycle phases, and the many kinds of AI out there?

# Which Goals States Pursue With Cooperative AI Governance

Scholars of IR and IPE have put forward different theories of international cooperation (Dai *et al.* 2010*)*. Viewed pragmatically, these approaches are neither entirely right or wrong, but capture different dynamics that are more or less pronounced in different policy fields and different geopolitical circumstances. To understand the varied motivations for states to cooperate on AI governance (Lemke *et al.* 2023), we first explore to what kind of governance challenges these theories typically apply, and what they suggest about the cooperation dynamics we should expect and that would be most apposite.

Our analysis builds upon previous work in global governance. Scholars of the field have long observed that global governance is difficult, partly because the multilateral institutions built by the United States and its partners after World War II largely omit rising powers (Patrick 2014), incentivizing these powers to either set up their own institutions (as China is doing with the proposed World Artificial Intelligence Cooperation Organization), engage in selective cooperation, or go it alone. AI has exacerbated the tendency to act unilaterally, as its general-purpose nature and the perception that there is an “AI race” make many countries skeptical vis-à-vis potentially constraining multilateral frameworks and premature regulation.

Moreover, there is no consensus on whether AI could pose a serious threat to the survival of humanity or not, which means that some countries believe it should be strictly regulated while other mainly see opportunity and want AI companies to charge ahead unconstrained (Büthe et al. 2022). Many policymakers highlight AI’s implications for competitiveness, complicating inter-country cooperation further. Against that background, we outline where global cooperation remains beneficial, and where selective cooperation is equally (or even more) effective. The first step in our analysis is to understand which cooperation patterns we can expect in different policy fields and under different geopolitical circumstances.

Neorealist IR scholarship holds that, because of the anarchic nature of the international system, states cooperate only when it helps them preserve or gain relative power (Waltz 2010). Thus, cooperation is driven by a desire to constrain rivals to prevent them from gaining a competitive edge. For example, the United States might support a global treaty banning autonomous weapons if doing so will prevent geopolitical rivals such as China from gaining a technological edge in their development. Since Neorealism also assumes that, due to the absence of a central authority above states, international institutions are weak and trust among states is low, there would have to be strong monitoring and enforcement mechanisms ensuring that countries stick to their commitments. In this zero-sum logic, cooperation is driven by narrowly defined self-interest and characterized by temporary alliances rather than deep, institutionalized cooperation.

Neoliberal institutionalism sees states cooperate because doing so helps them to manage the complex interdependence that arises from the transboundary nature of AI. For example, states might cooperate on rules for international data flows, AI-driven cyber threats, or global supply chain issues. Neoliberal institutionalism has particular purchase on issues for which cooperation benefits everyone (Keohane 2005). It assumes that repeated interactions and the establishment of institutions that help lower transaction costs, set expectations, and monitor compliance increase the chances of successful cooperation among states. Once such institutions are in place, states can work together on creating common standards, fostering data sharing, and preventing harmful uses of AI.

From a constructivist perspective, states cooperate because of shared ideas, identities, and norms (Wendt 1992). Thus, like-minded countries might work on joint AI governance frameworks that reflect values they hold in common or assumptions about AI threats that they might come to share—for example coalescing around worries about Artificial General Intelligence posing existential risks to humanity. Importantly, for constructivists, the “problems” addressed by shared AI governance frameworks are themselves constructed; how states perceive these problems shapes whether selective or global cooperation is most effective for solving them – whether, for example, potential productivity enhancements through AI use could benefit everyone, or whether they push countries into a zero-sum economic competition.

Finally, mercantilist IPE sees states cooperate to secure strategic industries, control technology value chains, or protect national champions (Drezner 2010). Cooperation becomes a tool of industrial policy, not just interdependence management. For example, the EU and the United States might align on AI standards to obstruct Chinese tech firms, preserve market dominance, and control data flows. AI governance then serves the goal of strategic decoupling or reshaping global capitalism. Mercantilist IPE provides a more structural and power-sensitive view of AI cooperation that looks at economic dominance, industrial rivalry, or inequality.

Taken together, this literature points to varied “intermediate goals” of cooperation that are more or less pronounced depending on the policy field and geopolitical context, which determine the ultimate goals of cooperation: building or bolstering geopolitical alliances; avoiding global arms races/races to the bottom; preventing the proliferation of harmful AI; realizing benefits from trade; sharing knowledge and best practices; promoting norms; and managing externalities of harmful AI and collective action problems. But to what degree are either selective or global forms of cooperation effective to tackle these, meaning that they have a high chance of countries actually attaining their intended goals? To answer that question, we explore these seven intermediate goals in a bit more detail.

These goals can clash in practice, not least because of AI’s general-purpose character. For example, a country might want to share knowledge regarding drones used to monitor construction sites but fear that such knowledge would also find military application. Depending on the geopolitical context, this problem could be insurmountable (if decision-makers care only about relative gains) or completely manageable (if decisionmakers perceive other countries as allies with low conflict potential). In concrete cases, many factors shape which perspective prevails. Here, however, we are interested in the problem-solving potential of different forms of cooperation, not in the concrete decisions of policymakers.

## Building or Bolstering Geopolitical Alliances

AI upsets existing international power constellations (Katagiri 2024), and by reshuffling offensive and defensive capabilities, it also affects the probability of war (Borchert *et al.* 2024). Moreover, alliances like NATO reassess their strategies for integrating AI into military operations (cf. NSCAI 2021). As the digital and material dimensions of warfare increasingly intermesh, allied countries face incentives to share information, mutually adapt systems, and trade in the relevant components to exploit economies of scale. While major AI powers (notably the NATO countries, China, and Russia) can forge alliances with other strategically positioned powers, countries that fall outside that category simply may have to bandwagon.

When it comes to bolstering geopolitical alliances, only selective cooperation makes sense. Note, however, that that applies only to those kinds of AI that have plausible geostrategic implications, such as military use, not to those without such implications. Moreover, the argument against broad and potentially global cooperation also does not hold when it concerns AI systems that have already been developed by geopolitical competitors, as well. The parallel with semiconductors is instructive: the United States has banned export of the most powerful computer chips to China. Such restrictions are less useful—and thus not observed in practice—for chips that China can also manufacture itself. Selective cooperation to build or bolster geopolitical alliances thus makes sense particularly with regard to cutting-edge technologies; less so for everything else.

## Avoiding Global Arms Races/Races to the Bottom

AI also generates prisoner’s-dilemma-style collective action problems.^[[2]](#footnote-2)^ A particularly vexing variant is the “race to the bottom”. In the regulatory version, countries may neglect AI safety and human rights protections in the hope that forbearance lets local companies thrive (Scholvin & Wigell 2018). The consequences of such competitive laxity can be dire: privacy infringements; premature release of biased systems; an unrestrained push for artificial general intelligence (Russell 2019; Tegmark 2017); and insufficient oversight of AI companies and the systems they are building. Cooperation can prevent such regulatory races to the bottom.

The most salient collective action problem concerns AI-powered warfare (Bode & Huelss 2022). With respect to LAWS, the US National Security Commission on AI (NSCAI) has explicitly argued that the United States cannot afford unilaterally to forgo such systems, given that enemy countries do not do so, either—a classic security dilemma (NSCAI 2021). Thus, to avoid arms races, only global agreements are useful. If even one country starts to develop AI-powered weapons, other countries have an incentive to follow suit.

## Preventing the Proliferation of Harmful AI

In theory, AI can be used to inflict various kinds of harm, for example through cyber-sabotage, easier access to destructive weapons, tools for espionage, and so on. Observers have therefore worried that harmful AI might end up in the wrong hands—for example, terrorists, geopolitical enemies or malicious political rulers, bent on oppressing local populations (Kreps 2021).

Preventing the proliferation of harmful AI requires pro-active collaboration, in which AI producers commit to restricting technology diffusion in ways that go beyond their narrow self-interest. As is true in other non-proliferation regimes, we can only expect stable and effective international arrangements if all major AI powers are involved. Global cooperation is a *sine qua non* to stem harmful AI diffusion effectively.

## Realizing Benefits from Trade

The enormous economies of scale in digital products and hence the low product unit costs mean that *ceteris paribus*, global trade should be welfare-enhancing. At the same time, large companies frequently benefit from the resulting oligopolistic tendencies (Open Markets Institute 2023) and amplify them by consciously limiting consumer choice, for example by locking consumers into proprietary digital ecosystems (Nitzberg & Zysman 2022; Srnicek 2017; Staab 2019). Big Tech exploitation of dominant market positions has triggered a wide backlash (for example, Durand 2020)—and thus also dented unrestricted trade in AI products.

In consequence, the relationship between Big Tech dominance and rule-based trade in AI products is more complicated than traditional free trade debates would suggest. International agreements could help attenuate these competitive dynamics, as they did, for example, in finance (Singer 2007). Inversely, domestic oligopolies might still recreate the undesirable effects of current cross-border ones (what Haggart & Tusikov 2023 call Digital Economic Nationalism). Moreover, governments, citizens, and consumers across the world already are customers of the largest AI-supplying companies, but then for products that originally did not rely on AI—think of Google (with Maps, search, Google Docs, etc.), Microsoft (Windows, Office, Azure), Amazon (retail), Apple (devices), and so on. As these companies intertwine AI with their other products, they make it difficult to sever AI trade ties selectively. In short, “limiting trade in AI” is more complex, and more difficult, than a simple assessment of who benefits and who does not would suggest.

Trade agreements could help ensure that foreign AI products and services conform to domestic standards and norms (Ulnicane 2022). When trade agreements counter oligopolistic tendencies and ensure that trade benefits are shared broadly, both selective and global cooperation can clearly be beneficial. Selective cooperation on trade can often be more easily achieved, but fairly managed global trade in principle has most to offer.

## Sharing Knowledge and Best Practices

Knowledge sharing in the AI field has two contradictory faces. The more governments see AI through a competitive lens, the less willing they may be to share expertise: why help others catch up, or get further ahead? At the same time, publicly sponsored academic research is widely accessible, also to competitors. And many forms of knowledge have limited competitive implications: innovations to make model training and inference less energy consuming or theoretical knowledge about new model architectures that are of little use without access to the right kind of data or computational resources, for example (Widder *et al.* 2023). The competitiveness implications of AI-relevant knowledge, in other words, vary widely, and so do the incentives to cooperate to share it on a reciprocal basis.

It is difficult for governments to develop effective AI regulation and enforcement, not least when it comes to the technical details, like safety assessments and bias identification—issues with which AI companies themselves still struggle (Balasubramaniam *et al.* 2023; Zuiderveen Borgesius 2018). That opens scope for cooperation, especially for countries with limited homegrown AI regulatory capabilities (Cafaggi & Pistor 2015) or capacities (Bach & Newman 2007; see also Lavenex *et al.* 2021). In instances in which best practice sharing is useful, it ideally happens globally. Where that is politically infeasible, selective cooperation in this domain is still useful.

## Promoting Norms

Democratic countries frequently warn against the dangers that AI poses to democracy, as they see it, and they push for international agreements to limit AI-powered mass surveillance, political repression, or the diffusion of political propaganda. The OECD, for example, with mostly functioning democracies as members, has been busy proselytizing for its AI Principles in the Arab Region, Africa, and South America (Russo & Oder 2023). Human rights protection and the promotion of democratic values in AI use and development are recurrent themes. The EU, too, hopes to diffuse AI Act-style rules to third countries (Almada & Radu 2024).

The motivations behind these initiatives can differ: democratic governments might well want to support people beyond their borders. They might also want to contain the further spread of digital authoritarianism and the potential loss of geopolitical allies or simply externalize their own rule sets for economic advantage (Bradford 2023). Here, we remain agnostic about what fuels the norm diffusion initiatives—how well-meaning or strategic they are. But international cooperation could, in theory, foster international alignment regarding AI ethics and promote consensus in this field. In norm promotion, both global and selective cooperation are very useful. If norms regarding ethical AI, for example, put at least some limits on the development and deployment of AI that violates human rights, every single person in any country who is covered by such norms counts as a “win.” Thus, even smaller-scale, selective cooperation is valuable for promoting norms (and perhaps more realistic than global cooperation on ethical AI standards, which is very difficult to achieve (Author, 2023)).

## Managing Externalities of Harmful AI and Collective Action Problems

Irrespective of the global dimension, much of the AI governance debate has concentrated on the specific kinds of harms AI systems may do (Crawford 2021; Dauvergne 2021). These harms from AI are thus a specific challenge this cluster of technologies constitutes (Smuha 2021), beyond the more generic ones such as sharing knowledge or avoiding regulatory races to the bottom.

Importantly for us, these AI harms can travel across borders. For example, AI’s environmental and climate impact can be enormous. Companies have frequently trained algorithms on copyrighted materials, also from abroad (cf. Haggart & Tusikov 2023). Democracy can suffer under the impact of AI-powered fake news and propaganda (Bridle 2018), and AI disrupts labor markets (Acemoglu & Johnson 2023; Frey 2019). Both dynamics can cut across borders, creating incentives for international cooperation, as those affected by the negative physical or policy externalities (Abbott & Snidal 2001) of other countries will want to cooperate with those countries to minimize externalities.

When it comes to addressing physical and policy externalities, global cooperation is most effective but selective cooperation can be useful as well. When the scope of the externality is itself not global, regional cooperation can be effective. However, even for global externalities, the scope of cooperation need not necessarily be global (Ostrom 2009)—agreements even among a smaller number of countries can attenuate competitive dynamics and allow them to pursue sustainability, for example.

Table 1 summarizes which kinds of cooperation are particularly useful for which kinds of governance challenges. The notion that only like-minded countries can (or should) cooperate on AI governance is just as misguided as the notion that AI governance needs to be global. Depending on the goals cooperation is meant to advance, global cooperation can be more useful than selective cooperation (or even the only viable option, e.g., when trying to forestall the proliferation of harmful AI to malicious actors); selective and global cooperation can be equally useful; or selective cooperation can be more useful. Crucially, there is no reason that countries cannot cooperate on some aspects of AI governance and not on others.

|  | *Selective cooperation* | *Global cooperation* |
| --- | --- | --- |
| Building geopolitical alliances | ++ | - |
| Preventing proliferation of harmful AI | - | ++ |
| Realizing benefits from trade | ++ | ++ |
| Sharing knowledge and best practices | + | ++ |
| Promoting norms | ++ | ++ |
| Managing externalities of harmful AI and collective action problems | + | ++ |

*Table 1. Utility of selective versus global cooperation in AI governance. (++ = very useful, + = somewhat useful, - = not useful)*

# Externalities of harmful AI across lifecycle phases and types of AI

Many technologies can generate harms that travel across borders, for example in the form of environmental damage. How those can be tackled effectively depends on the technologies in question and the specific harms they create. We therefore zoom in on the last row of Table 1—managing externalities of harmful AI and collective action problems—to understand the governance challenges and scope of required cooperation in more detail. To do so, we add two axes of distinction: different phases of the AI lifecycle, and different types of AI.

## Development versus deployment

For our purposes, the AI lifecycle can be usefully disaggregated into two main different phases: system development and system deployment (Burr & Leslie 2023; De Silva & Alahakoon 2022). For us, the development phase includes the selection of an appropriate algorithm, tested on the dataset and fine-tuned and validated along the way. From a governance perspective, the fine-tuning of foundation models is an especially important part of the development phase, as fine-tuning is “where alignment often happens” (Ohm 2024, p. 224). In other words, fine-tuning allows anyone who can access a pre-trained model, and who has a dataset and some technical knowledge, to adjust the foundation model in order to align with their desired values (Ohm 2024, p. 228). This means public policymakers (or those to whom they outsource this process) have a unique opportunity to shape foundation models through finetuning. During the deployment phase, the trained model is integrated into a target production system. Key goals during this phase include ensuring that the model can be effectively scaled to different workloads and datasets, training the AI system’s userbase on how to operate the model, monitoring the model and, if needed, updating it.

These two phases raise different governance issues, so that the salience of the policy concerns identified above varies throughout the AI lifecycle. In the development phase, questions about the data used to train the algorithm and about the unbiasedness, transparency, and fairness of the system being built are particularly pressing (Gasser & Mayer-Schönberger 2024). During the deployment phase, concerns about use cases weigh more heavily (Smith & Browne 2019).

Governments have different degrees of leverage over the development and deployment phases of the AI life cycle. As cutting-edge AI originates from a small number of places, most countries will have little leverage over AI development. If anything, they can ask companies developing foundation models to finetune the pre-trained base models to comply with local rules and regulations. However, foreign companies may decide to ignore local requirements and simply withhold products from markets altogether. Even where they do agree to host country rules, compliance may be hard to monitor. Aligning requirements with countries that actually develop AI then makes sense—if and when it is achievable.

In contrast, governments should have more leverage over AI deployment. When the users of AI systems are large organizations, like big companies or public bodies, governments can regulate use cases relatively easily and thereby steer how AI is deployed within their jurisdiction. That becomes more difficult when individuals or small organizations are the end users, because in some instances they can access AI-powered services provided from abroad through a VPN client. Table 2 gives examples of AI applications that fall into the different categories.

|  | Harm *with* cross-border effects, incentivizing cooperation | Harms *without* cross-border effects, incentivizing *no* cooperation |
| --- | --- | --- |
| AI *development* | Development of foundation models that might pose existential risk | Development of biased algorithms in public administration |
| AI *deployment* | Climate impact of excessive energy use of AI systems | Use of AI for excessive surveillance of public space |

*Table 2: Examples of AI development and deployment with varying incentives for cooperation*

## Governance challenges across types of AI

Having laid out potential motivations for AI governance cooperation across the development and deployment phases, we now examine the different governance issues raised by different kinds of AI and analyze whether they call for selective or global cooperation. After all, AI technologies can be applied across fields and industries, with countless use cases (Brynjolfsson *et al.* 2021), which makes “cooperation in AI governance” a dubiously broad field. An AI-enabled vacuum cleaner raises very different governance problems than an AI-powered drone for the battlefield. Some of these problems can best be addressed through selective agreement; others require global cooperation. In the household appliance example, countries that want to trade with each other would need shared safeguards against excessive personal data collection by manufacturers as a precondition for cross-border sales. In the case of AI-powered drones, in contrast, the goals would be to counter excessive proliferation to third countries and malevolent actors, and to avoid an arms race among drone-developing countries—goals that can only be achieved through global cooperation.

These examples show how different kinds of AI incentivize different scopes of interstate cooperation. The systems under the AI heading can be sliced and diced in different ways (Spector *et al*. 2022). Rather than canvassing AI technologies as a whole (a questionable enterprise to begin with), we therefore examine four different kinds of AI as examples that raise divergent concerns and thereby divergent cooperation incentives: foundation models, AI-powered physical products, small-scale AI as a service, and militarily relevant AI.

Even these four kinds, however, are not mutually exclusive; foundation models, for example, are also software than can be accessed remotely (“as a service”). And different AI systems and model architectures are increasingly combined in real-world applications (Masley *et al.* 2024). That said, these four categories do cover central AI applications and use cases, also as they feature in contemporary political debate. That gives them analytical leverage as we show just how diverse their implications for international cooperation are.

### Foundation Models

AI foundation models are a class of AI models characterized by their vast size, extensive training data, and general-purpose nature (for example, Moës & Ryan 2023). That distinguishes them from other ML models, which typically perform narrower tasks such as process optimization, image classification, or trend forecasting (Mitchell 2019).

Foundation models are the basis for specialized downstream applications such as chatbots. This versatility presents large challenges for policymakers (Seger *et al.* 2023). For example, malevolent users might tweak a model to bypass built-in safeguards, say to extract instructions to make explosives. At the same time, as models integrate text, sound, video and other content, they may inch closer towards artificial general intelligence. They are likely still far off. But the potential quandaries are clear and serious enough to warrant dedicated attention (Bostrom 2014; Russell 2019; Schneider 2019).

What do these characteristics imply for cooperation in governance across the two lifecycle phases? The deployment of foundation models can in principle be regulated unilaterally. The development of large models, in contrast, is harder to steer if they are built abroad. Given the enormous cost of training the largest models (Vipra & Myers West 2023), AI companies may refuse to customize them to each country’s wishes. If anything, they might fine-tune foundation models to comply with local requirements (Ohm 2024). If countries were to cooperate and confront foundation model builders with a single set of demands, the latter would be much more likely simply to heed them in model development.

For remotely accessible foundation models, large gaps between domestic and foreign development rules matter: most governments must either import what others build or go without them altogether (Author, 2024b). By contrast, differences in how models are used abroad matter less; even objectionable uses (e.g., AI-enabled domestic surveillance) don’t directly create cross-border externalities.

### AI-powered Physical Products

Consider next a completely different category of AI systems: physical products whose functionality depends on AI—modern cars or kitchen appliances, for example, but also medical or communication devices that may malfunction when you really cannot afford it. Here safety clearly is an issue, as is resilience against cyberattacks and potentially excessive data collection by device manufacturers.

How do cooperation incentives look for this category of AI systems? As was true for foundation models, jurisdictions can in principle set local rules for system deployment. In contrast to the foundation models, however, these devices typically come with simpler systems built into them. That makes it possible for companies to finetune systems to local demands at relatively little cost—think of integrated privacy safeguards, for example. In consequence, for these types of products, the incentive to cooperate is lower in the development phase than for foundation models.

For AI-enabled physical products, divergence between domestic and foreign rules matters less. Governments can require imported products to meet local specifications—baked in during development—and block non-compliant imports. Such restrictions are most relevant for high-risk items (e.g., autonomous vehicles, AI-driven medical devices); for low-risk goods (e.g., wearables, smart home devices), cooperation often defaults to adopting the exporter’s standards.

### Small-scale AI as a Service

AI is integrated into many different kinds of products that are available online. In a crucial difference to the previous category, the products in question are not built into specific devices—such as a vacuum cleaner—but can be transferred digitally to the end user or accessed remotely.

This digital character means that it is inherently difficult to keep unwanted software outside a jurisdiction; regulatory interdependence in inevitably higher (Author 2024b). Just think of digital personal assistants, recommender systems used for content feed personalization, image recognition software, or websites that offer deepfake videos—all of which may compromise user safety or privacy. Effective control over such applications’ availability within a country’s borders can require substantial cooperation.

This category overlaps with foundation models but we distinguish between large models owned by a few prominent firms and simpler tools offered by many providers (e.g., voice cloning, deepfakes). Because these tools are remotely accessible, cooperation centers on how they are provided and used—that is, deployment. The aim is to restrict availability so deployment can be coordinated and controlled across borders. As before, the cooperation incentive is highest for high-risk systems (e.g., AI surveillance, autonomous trading) and much lower for low-risk ones (e.g., customer-service chatbots, translation).

For small-scale AI as a service, differences between domestic development rules and those abroad do not matter a lot. In principle, the specifications of small-scale AI as a service can easily be changed to conform with the preferences of countries importing these services. However, divergent deployment rules abroad matter because they affect to whom, and under which circumstances, these systems are available.

### Militarily Relevant AI

AI is transforming modern military operations in numerous ways, ranging from autonomous systems (Firlej & Taeihagh 2021) to decision-making aids (Borchert *et al.* 2024). While AI enhances operational capabilities, its integration into military systems introduces ethical quandaries, such as loss of human oversight, escalation of conflict, and issues of accountability and transparency (Taddeo *et al.* 2021). These concerns incentivize governments to cooperate in order to try to establish international frameworks that regulate both the development and deployment of AI-powered military equipment in international contexts, akin to international rules of war more generally. One key challenge for such cooperation is the fact that once one country starts developing AI-powered weapons, a security dilemma ensues in which other countries are tempted to develop similar weapons in response (Booth & Wheeler 2023). Hence the only form of cooperation that works here is truly global cooperation, including at least all major military powers. However, such global cooperation is difficult to achieve, as each country individually has an incentive to not cooperate even if all countries would be better off if everyone did—a classic prisoner’s dilemma.

When it comes to militarily relevant AI, it matters a lot whether domestic development and deployment rules differ from those abroad. Foreign development rules determine whether AI-powered weapons, for example, are fully autonomous or keep a human in the loop in a meaningful way. This makes a difference in warfare (Firlej & Taeihagh 2021). And since militarily relevant AI is not usually meant to be deployed locally (unless there is a civil war or government repression), the deployment rules of countries that produce militarily relevant AI have serious, tangible consequences for other countries. Table 3 summarizes the cross-border impact of domestic regimes governing the development and deployment of the four different kinds of AI identified above.

|  |  | Divergence between domestic AI *development* rules and those abroad… | |
| --- | --- | --- | --- |
|  |  | …matters a lot, making cooperation on AI development desirable. | …does not matter a lot, making cooperation on AI development optional. |
| Divergence between domestic AI *deployment* rules and those abroad… | …matters a lot, making cooperation on AI development desirable. | Military AI | Small-scale AI as a service |
|  | …does not matter a lot, making cooperation on AI development optional. | Foundation models | AI-powered physical products |

*Table 3. Cross-border impact of domestic regimes for AI governance*

Having identified four different kinds of AI that engender different policy concerns, we can now map them onto the different phases of the AI lifecycle to see which concerns are most pressing in which phase and build a systematic understanding of when and how countries might want to cooperate to address these concerns effectively. Table 4 below integrates our previous arguments about whether selective or global cooperation are most useful to address governance issues raised by the four exemplary kinds of AI across the lifecycle. We also added our analysis of how useful it is to unilaterally regulate foreign AI during the two lifecycle phases.

|  | *Unilateral regulation of foreign AI* | | *Selective cooperation* | | *Global cooperation* | |
| --- | --- | --- | --- | --- | --- | --- |
|  | *Development* | *Deployment* | *Development* | *Deployment* | *Development* | *Deployment* |
| Foundation models | - | + | + | - | ++ | - |
| AI-powered physical products | - | + | + | - | ++ | - |
| Small-scale software as a service | - | + | + | - | ++ | - |
| Militarily relevant AI | - | - | + | + | ++ | ++ |

*Table 4. Relative benefits of different scopes of cooperation to avoid harms specific to AI types, split across development and deployment phases of the AI life cycle (++ = very useful, + = somewhat useful, - = not useful)*

As summed up in Table 4, in the case of non-military AI, wide-ranging international collaboration on development is desirable (the wider, the better), while deployment is best governed primarily at the national level. The notable exception is military AI, where deployment likewise warrants collectively formulated rules to preclude arms races and associated collective-action failures. Overall, the gross benefits of global cooperation typically outstrip those of selective cooperation. However, once we consider the net benefits – so discounting for the costs of cooperation, which rise with the number of actors involved – the picture is more mixed, with global cooperation coming out on top in some instances and selective cooperation in others.

An example helps to flesh out the proposed approach. Imagine a chatbot that generates political propaganda in response to questions, for example about Russia’s war on Ukraine—whether intentionally or not. Policymakers from a Western liberal democracy (country A) might want to prevent such a chatbot from being developed. But that may be difficult if the company behind it is from another country (B). Thus, country A will want to work with country B to clamp down on the propaganda-spreading chatbot. Ideally, country A would want a global agreement that prevents such chatbots from being made *anywhere*. Given different legal systems and interests around the world, that may be difficult, however. Selective cooperation then is the second best but still very useful option if country B and other prominent partner countries are included. If agreement with country B is impossible, country A can still try to enforce local finetuning of the chatbot or forbid its deployment altogether – both difficult and depending on the company’s willingness to cooperate. Global agreement against such chatbots may thus be preferrable but should the costs to reach such agreement be too high, selective cooperation with the countries that matter to domestic chatbot use in country A may be most beneficial in net terms.

# Conclusion

Just about any expert in the field agrees that there is something to be won in international cooperation on AI. But how, and with whom? To our mind, too much of the debate falls into clear camps: either cooperation should be global (which normally means including Europe, the United States, and certainly China, too), or it should be selective, involving “like-minded countries” (so excluding China).

Our analysis suggests that that view is too simplistic. Incentives for cooperation vary, and they may point in different directions. For some aspects of AI policy, global cooperation is the only approach that works; for others, selective cooperation is a useful second-best or even an equally worthwhile solution. That means that governments should not approach global and selective cooperation as either/or alternatives. Instead, a variable geometry of cooperation on AI matters is not only possible, but indeed a desirable approach to such a broad field with such diverse concerns.

On the one hand, then, the “we need global cooperation”-camp should realize that, desirable as such encompassing agreements would be, more circumscribed cooperations may deserve their support, as well. If those are the only constellations that work, then so be it.

On the other hand, and more importantly to our mind, there is no reason why AI cooperation should completely follow an alliance-building logic. The contemporary geo-economic competition between China and the United States is a political reality. It would take more than successful AI cooperation to supersede that antagonism. But that does not mean that there would not be many areas in which cooperation could be beneficial to both sides—think of avoiding arms races, forestalling the unchecked proliferation of dangerous AI to terrorists and other criminals, creating basic safety standards for AI-powered consumer appliances, and so on. Several cooperation logics can exist side by side.

By the same token, there is no reason why the EU—while clearly a longstanding ally of the United States—should slavishly follow the American lead on AI cooperation. There are forms of knowledge sharing, trade in non-sensitive products, ethics discussions, safety research and so on that could be beneficially pursued along a Sino-European axis even if the United States were not involved.

For further research, it would be fruitful to analyze whether and how these cooperation logics play out in practice. There is lively debate about the degree to which the EU and the United States should align AI governance with each other and how much scope there is for cooperation with China. But how do these three powers (and others) actually behave? There is a multitude of different AI governance initiatives underway in global and regional forums. Examining which of the imperatives we have outlined here actually prevail in practice, and why, will be a worthwhile next step.

**Funding Statement**

This research was supported by the Dutch Science Council (NWO) through a Vici grant [VI.C.211.032].

**References**

Abbott, Kenneth W., and Duncan Snidal. 2001. ‘International “standards” and International Governance’. *Journal of European Public Policy* 8(3): 345–70. doi:10.1080/13501760110056013.

Acemoglu, Daron, and Simon Johnson. 2023. *Power and Progress: Our Thousand-Year Struggle Over Technology and Prosperity*. New York: PublicAffairs.

Ala-Pietilä, Pekka, and Nathalie A. Smuha. 2021. ‘A Framework for Global Cooperation on Artificial Intelligence and Its Governance’. In *Reflections on Artificial Intelligence for Humanity*, eds Bertrand Braunschweig and Malik Ghallab. Cham: Springer International Publishing, 237–65. doi:10.1007/978-3-030-69128-8_15.

Allen, Gregory C. 2022. ‘One Key Challenge for Diplomacy on AI: China’s Military Does Not Want to Talk’. https://www.csis.org/analysis/one-key-challenge-diplomacy-ai-chinas-military-does-not-want-talk (March 27, 2024).

Almada, Marco, and Anca Radu. 2024. ‘The Brussels Side-Effect: How the AI Act Can Reduce the Global Reach of EU Policy’. *German Law Journal*: 1–18. doi:10.1017/glj.2023.108.

Bach, David, and Abraham L. Newman. 2007. ‘The European Regulatory State and Global Public Policy: Micro-Institutions, Macro-Influence’. *Journal of European Public Policy*. doi:10.1080/13501760701497659.

Balasubramaniam, Nagadivya, Marjo Kauppinen, Antti Rannisto, Kari Hiekkanen, and Sari Kujala. 2023. ‘Transparency and Explainability of AI Systems: From Ethical Guidelines to Requirements’. *Information and Software Technology* 159: 107197. doi:10.1016/j.infsof.2023.107197.

‘Basics of IAEA Safeguards’. 2016. http://www.iaea.org/topics/basics-of-iaea-safeguards (January 22, 2026).

Beaumier, Guillaume, Kevin Kalomeni, Malcolm Campbell-Verduyn, Marc Lenglet, Serena Natile, Marielle Papin, Daivi Rodima-Taylor, Arthur Silve, and Falin Zhang. 2020. ‘Global Regulations for a Digital Economy: Between New and Old Challenges’. *Global Policy* 11(4): 515–22. doi:10.1111/1758-5899.12823.

Bode, Ingvild, and Hendrik Huelss. 2022. *Autonomous Weapons Systems and International Norms*. Montreal: McGill University Press.

Booth, Ken, and Nicholas J. Wheeler. 2023. ‘Uncertainty’. In *Security Studies*, Routledge.

Borchert, Heiko, Torben Schütz, and Jospeh Verbovsky. 2024. *The Very Long Game. 25 Case Studies on the Global State of Defense AI*. Cham: Springer Nature.

Bostrom, Nick. 2014. *Superintelligence. Paths, Dangers, Strategies*. Oxford: Oxford University Press.

Bradford, Anu. 2023. *Digital Empires: The Global Battle to Regulate Technology*. Oxford University Press.

Bridle, James. 2018. *New Dark Age. Technology and the End of the Future*. London: Verso.

Brynjolfsson, Erik, Daniel Rock, and Chad Syverson. 2021. ‘The Productivity J-Curve: How Intangibles Complement General Purpose Technologies’. *American Economic Journal: Macroeconomics* 13(1): 333–72. doi:10.1257/mac.20180386.

Burr, Christopher, and David Leslie. 2023. ‘Ethical Assurance: A Practical Approach to the Responsible Design, Development, and Deployment of Data-Driven Technologies’. *AI and Ethics* 3(1): 73–98. doi:10.1007/s43681-022-00178-0.

Büthe, Tim, Christian Djeffal, Christoph Lütge, Sabine Maasen, and Nora von Ingersleben-Seip. 2022. ‘Governing AI – Attempting to Herd Cats? Introduction to the Special Issue on the Governance of Artificial Intelligence’. *Journal of European Public Policy* 29(11): 1721–52. doi:10.1080/13501763.2022.2126515.

Büthe, Tim, and Walter Mattli. 2011. ‘The New Global Rulers: The Privatization of Regulation in the World Economy’. In *The New Global Rulers*, Princeton University Press. doi:10.1515/9781400838790.

Cafaggi, Fabrizio, and Katharina Pistor. 2015. ‘Regulatory Capabilities: A Normative Framework for Assessing the Distributional Effects of Regulation’. *Regulation & Governance* 9(2): 95–107. doi:10.1111/rego.12065.

Cheng, Jing, and Jinghan Zeng. 2023. ‘Shaping AI’s Future? China in Global AI Governance’. *Journal of Contemporary China* 32(143): 794–810. doi:10.1080/10670564.2022.2107391.

Cordell, Kristen. 2020. ‘The International Telecommunication Union: The Most Important UN Agency You Have Never Heard Of’. https://www.csis.org/analysis/international-telecommunication-union-most-important-un-agency-you-have-never-heard (March 27, 2024).

Crawford, Kate. 2021. *The Atlas of AI: Power, Politics, and the Planetary Costs of Artificial Intelligence*. Yale University Press.

Dai, Xinyuan, Duncan Snidal, and Michael Sampson. 2010. ‘International Cooperation Theory and International Institutions’. In *Oxford Research Encyclopedia of International Studies*, doi:10.1093/acrefore/9780190846626.013.93.

Dauvergne, Peter. 2021. ‘The Globalization of Artificial Intelligence: Consequences for the Politics of Environmentalism’. *Globalizations* 18(2): 285–99. doi:10.1080/14747731.2020.1785670.

De Silva, Daswin, and Damminda Alahakoon. 2022. ‘An Artificial Intelligence Life Cycle: From Conception to Production’. *Patterns* 3(6): 100489. doi:10.1016/j.patter.2022.100489.

Drezner, Daniel W. 2010. ‘Mercantilist and Realist Perspectives on the Global Political Economy’. In *Oxford Research Encyclopedia of International Studies*, doi:10.1093/acrefore/9780190846626.013.260.

Durand, Cédric. 2020. *Techno-Féodalisme - Critique de l’économie Numérique*. Paris: Zones. La Découverte.

Firlej, Mikolaj, and Araz Taeihagh. 2021. ‘Regulating Human Control over Autonomous Systems’. *Regulation & Governance* 15(4): 1071–91. doi:10.1111/rego.12344.

Frey, Carl Benedict. 2019. *The Technology Trap: Capital, Labor, and Power in the Age of Automation*. Princeton: Princeton University Press.

Gasser, Urs, and Viktor Mayer-Schönberger. 2024. *Guardrails. Guiding Human Decisions in the Age of AI*. Princeton: Princeton University Press.

Global Partners Digital. 2024. ‘The Final Report of HLAB-AI: Our Analysis and Thoughts – Global Partners Digital’. https://www.gp-digital.org/news/the-final-report-of-hlab-ai-our-analysis-and-thoughts/ (April 1, 2025).

Haggart, Blayne, and Natasha Tusikov. 2023. *The New Knowledge. Information, Data and the Remaking of Global Power*. Lanham: Rowman & Littlefield.

Hitzler, Pascal, Aaron Eberhart, Monireh Ebrahimi, Md Kamruzzaman Sarker, and Lu Zhou. 2022. ‘Neuro-Symbolic Approaches in Artificial Intelligence’. *National Science Review* 9(6): nwac035. doi:10.1093/nsr/nwac035.

Hoos, Holger, and Morten Irgens. 2023. ‘Viewpoint: Europe Needs a CERN for Artificial Intelligence | Science|Business’. https://sciencebusiness.net/viewpoint/ai/viewpoint-europe-needs-cern-artificial-intelligence (April 2, 2025).

House, The White. 2021. ‘Remarks by President Biden at the 2021 Virtual Munich Security Conference’. *The White House*. https://www.whitehouse.gov/briefing-room/speeches-remarks/2021/02/19/remarks-by-president-biden-at-the-2021-virtual-munich-security-conference/ (March 27, 2024).

von Ingersleben-Seip, Nora. 2023. ‘Competition and Cooperation in Artificial Intelligence Standard Setting: Explaining Emergent Patterns’. *Review of Policy Research* 40(5): 781–810. doi:10.1111/ropr.12538.

International Telecommunication Union (ITU). 2024. ‘Key Findings on the State of Global AI Governance’. *ITU*. https://www.itu.int/hub/2024/07/key-findings-on-the-state-of-global-ai-governance/ (August 21, 2024).

Justo-Hanani, Ronit. 2022. ‘The Politics of Artificial Intelligence Regulation and Governance Reform in the European Union’. *Policy Sciences* 55(1): 137–59. doi:10.1007/s11077-022-09452-8.

Katagiri, Nori. 2024. ‘Artificial Intelligence and Cross-Domain Warfare: Balance of Power and Unintended Escalation’. *Global Society* 38(1): 34–48. doi:10.1080/13600826.2023.2248179.

Keohane, Robert O. 2005. *After Hegemony: Cooperation and Discord in the World Political Economy*. Princeton University Press.

Kerry, Cameron F. 2024. ‘The Good, the Not-so-Good, and the Ugly of the UN’s Blueprint for AI’. *Brookings*. https://www.brookings.edu/articles/the-good-the-not-so-good-and-the-ugly-of-the-uns-blueprint-for-ai/ (September 17, 2024).

Kerry, Cameron F., Joshua P. Meltzer, Andrea Renda, and Andrew W. Wyckoff. 2024. ‘Should the UN Govern Global AI?’ *Brookings*. https://www.brookings.edu/articles/should-the-un-govern-global-ai/ (August 19, 2024).

Kratsios, Michael. 2025. ‘Remarks at the Security Council’s Open Debate on Artificial Intelligence and International Peace and Security’. https://usun.usmission.gov/remarks-at-the-security-councils-open-debate-on-artificial-intelligence-and-international-peace-and-security/ (October 2, 2025).

Kreps, Sarah. 2021. *Democratizing Harm: Artificial Intelligence in the Hands of Nonstate Actors*. Washington DC: Brookings Institution.

Lavenex, Sandra, Omar Serrano, and Tim Büthe. 2021. ‘Power Transitions and the Rise of the Regulatory State: Global Market Governance in Flux’. *Regulation & Governance* 15(3): 445–71. doi:10.1111/rego.12400.

Lemke, Nicole, Philipp Trein, and Frédéric Varone. 2023. ‘Agenda-Setting in Nascent Policy Subsystems: Issue and Instrument Priorities across Venues’. *Policy Sciences* 56(4): 633–55. doi:10.1007/s11077-023-09514-5.

Masley, Nestor, Fattorini, Loredana, Raymond Perrault, Vanessa Parli, Anka Reuel, Erik Brynjolfsson, John Etchemendy, et al. 2024. *The AI Index 2024 Annual Report*. Stanford: Stanford University. https://aiindex.stanford.edu/report/.

Mitchell, Melanie. 2019. *Artificial Intelligence. A Guide for Thinking Humans*. London: Pelican Books.

Moës, Nicolas, and Frank Ryan. 2023. *Heavy Is the Head the Wears the Crown*.

Mueller, Milton. 2024. ‘Rethinking AI Governance: The Political Economy of the Digital Ecosystem’. https://papers.ssrn.com/abstract=4911002 (November 11, 2024).

Nitzberg, Mark, and John Zysman. 2022. ‘Algorithms, Data, and Platforms: The Diverse Challenges of Governing AI’. *Journal of European Public Policy*: 1–26. doi:10.1080/13501763.2022.2096668.

NSCAI. 2021. ‘Final Report by the National Security Commission on AI’. https://reports.nscai.gov/final-report/introduction.

Ohm, Paul. 2024. ‘Focusing On Fine-Tuning: Understanding The Four Pathways For Shaping Generative AI’. *Science and Technology Law Review* 25(2). doi:10.52214/stlr.v25i2.12762.

Open Markets Institute. 2023. *AI in the Public Interest: Confronting the Monopoly Threat*. Washington DC.

Ostrom, Elinor. 2009. ‘A Polycentric Approach for Coping with Climate Change’. doi:10.2139/ssrn.1934353.

Patrick, Stewart. 2014. ‘The Unruled World: The Case for Good Enough Global Governance Essay’. *Foreign Affairs* 93(1): 58–73.

Paul, Regine. 2023. ‘European Artificial Intelligence “Trusted throughout the World”: Risk-Based Regulation and the Fashioning of a Competitive Common AI Market’. *Regulation & Governance*: rego.12563. doi:10.1111/rego.12563.

Politi, Daniel. 2021. ‘U.S. Has Lost AI Race to China, According to Former Software Chief at Pentagon’. *Slate*. https://slate.com/news-and-politics/2021/10/us-lost-artificial-intelligence-race-china-nicolas-chaillan.html (March 27, 2024).

Russell, Stuart. 2019. *Human Compatible. Artificial Intelligence and the Problem of Control*. New York: Viking.

Russo, Lucia, and Noah Oder. 2023. ‘How Countries Are Implementing the OECD Principles for Trustworthy AI’. https://oecd.ai/en/wonk/national-policies-2 (August 21, 2024).

Schneider, Susan. 2019. *Artificial You. AI and the Future of Your Mind*. Princeton: Princeton University Press.

Scholvin, Sören, and Mikael Wigell. 2018. ‘Power Politics by Economic Means: Geoeconomics as an Analytical Approach and Foreign Policy Practice’. *Comparative Strategy* 37(1): 73–84. doi:10.1080/01495933.2018.1419729.

Searle, John R. 1995. *The Construction of Social Reality*. New York: Free Press.

Singer, David. 2007. *Regulating Capital. Setting Standards for the International Financial System*. Ithaca: Cornell University Press.

Smith, Brad, and Carol Ann Browne. 2019. *Tools and Weapons. The Promise and Peril of the Digital Age*. London: Penguin.

Smuha, Nathalie. 2021. ‘Beyond the Individual: Governing AI’s Societal Harm’. *Internet Policy Review* 10(3): 1–32.

Snidal, Duncan. 1985. ‘Coordination versus Prisoners’ Dilemma: Implications for International Cooperation and Regimes’. *American Political Science Review* 79(4): 923–42. doi:10.2307/1956241.

Solomon, Jay. 2023. ‘Ex-Pentagon Chief: US, China Locked in Existential Struggle for AI Dominance | Semafor’. https://www.semafor.com/article/07/27/2023/us-china-existential-struggle-ai-dominance (March 27, 2024).

Spector, Alfred, Peter Norvig, Chris Wiggins, and Jeanette Wing. 2022. *Data Science in Context. Foundations, Challenges, Opportunities*. Cambridge: Cambridge University Press.

Srnicek, Nick. 2017. *Platform Capitalism*. Cambridge: Polity Press.

Staab, Philipp. 2019. *Digitaler Kapitalismus. Markt Und Herrschaft in Der Ökonomie Der Unknappheit*. Frankfurt am Main: Suhrkamp.

Taddeo, Mariarosaria, David McNeish, Alexander Blanchard, and Elizabeth Edgar. 2021. ‘Ethical Principles for Artificial Intelligence in National Defence’. *Philosophy & Technology* 34(4): 1707–29. doi:10.1007/s13347-021-00482-3.

Taeihagh, Araz. 2021. ‘Governance of Artificial Intelligence’. *Policy and Society* 40(2): 137–57. doi:10.1080/14494035.2021.1928377.

Tegmark, Max. 2017. *Life 3.0. Being Human in the Age of Artificial Intelligence*. London: Penguin.

Ulnicane, Inga. 2022. ‘Artificial Intelligence in the European Union’. In *The Routledge Handbook of European Integrations*, Taylor & Francis. doi:10.4324/9780429262081-19.

Ulnicane, Inga. 2024. ‘Governance Fix? Power and Politics in Controversies about Governing Generative AI’. *Policy and Society*: puae022. doi:10.1093/polsoc/puae022.

United Nations High-Level Advisory Body on Artificial Intelligence. 2024. *Govering AI for Humanity: Final Report*. New York, NY: United Nations.

Vipra, Jai, and Sarah Myers West. 2023. *Computational Power and AI*. New York. https://ainowinstitute.org/publication/policy/compute-and-ai.

Waltz, Kenneth N. 2010. *Theory of International Politics*. Waveland Press.

Wendt, Alexander. 1992. ‘Anarchy Is What States Make of It: The Social Construction of Power Politics’. *International Organization* 46(2): 391–425.

Widder, David Gray, Sarah West, and Meredith Whittaker. 2023. ‘Open (For Business): Big Tech, Concentrated Power, and the Political Economy of Open AI’. doi:10.2139/ssrn.4543807.

Yi, Wang. 2024. ‘Wang Yi on Global AI Governance: Ensure That AI Is a Force for Good, Ensure Safety and Ensure Fairness’. https://www.mfa.gov.cn/mfa_eng/wjbzhd/202403/t20240308_11256430.html (March 27, 2024).

Zuiderveen Borgesius, Frederik. 2018. *Discrimination, Artificial Intelligence, and Algorithmic Decision-Making*. Directorate General of Democracy, Council of Europe.

1. It is noteworthy, however, that in its final report, the UN AI Advisory Body walked back from this recommendation and instead suggested a “light-touch” AI Office within the UN Secretariat that would serve as a coordinating mechanism for existing AI governance initiatives (United Nations High-Level Advisory Body on Artificial Intelligence 2024). [↑](#footnote-ref-1)
2. In game theory, a prisoner’s dilemma game is one in which non-cooperation is the indivually rational choice for each of the players even though cooperation would yield higher payoffs for each (e.g., Snidal 1985). [↑](#footnote-ref-2)
